# Supplementary material for: Neuronal mitochondrial morphology is significantly affected by both fixative and oxygen level during perfusion
Source: Front Mol Neurosci. 2022 Nov 2;15:1042616. doi: 10.3389/fnmol.2022.1042616 (PMC9667081; doi:10.3389/fnmol.2022.1042616)
Supplement: Supplementary Table 1 — Fixation methods and outcomes. A table listing the fixation conditions and their outcomes for both in vitro and in vivo conditions in the paper. [file Data_Sheet_1.pdf]

Supplementary Table 1

	Fixative (in PBS)	Methods	Fragmentation
In vitro	2% PFA/0.075% GA (4% PFA/4% Sucrose*)	Direct fixation with fixative for 10-15min (Brief rinse with DPBS before fixation*)	-
	4% PFA		↑
In vivo	2% PFA/0.075% GA (4% PFA**)	Anesthesia with oxygen	-
		Anesthesia without oxygen (hypoxic)	↑
		Direct perfusion with fixative	-
		DBPS pre-flush before fixation	↑

*Can be used as an alternative option.

***In vivo* application of 4% PFA with optimized methods conserves mitochondrial shapes.

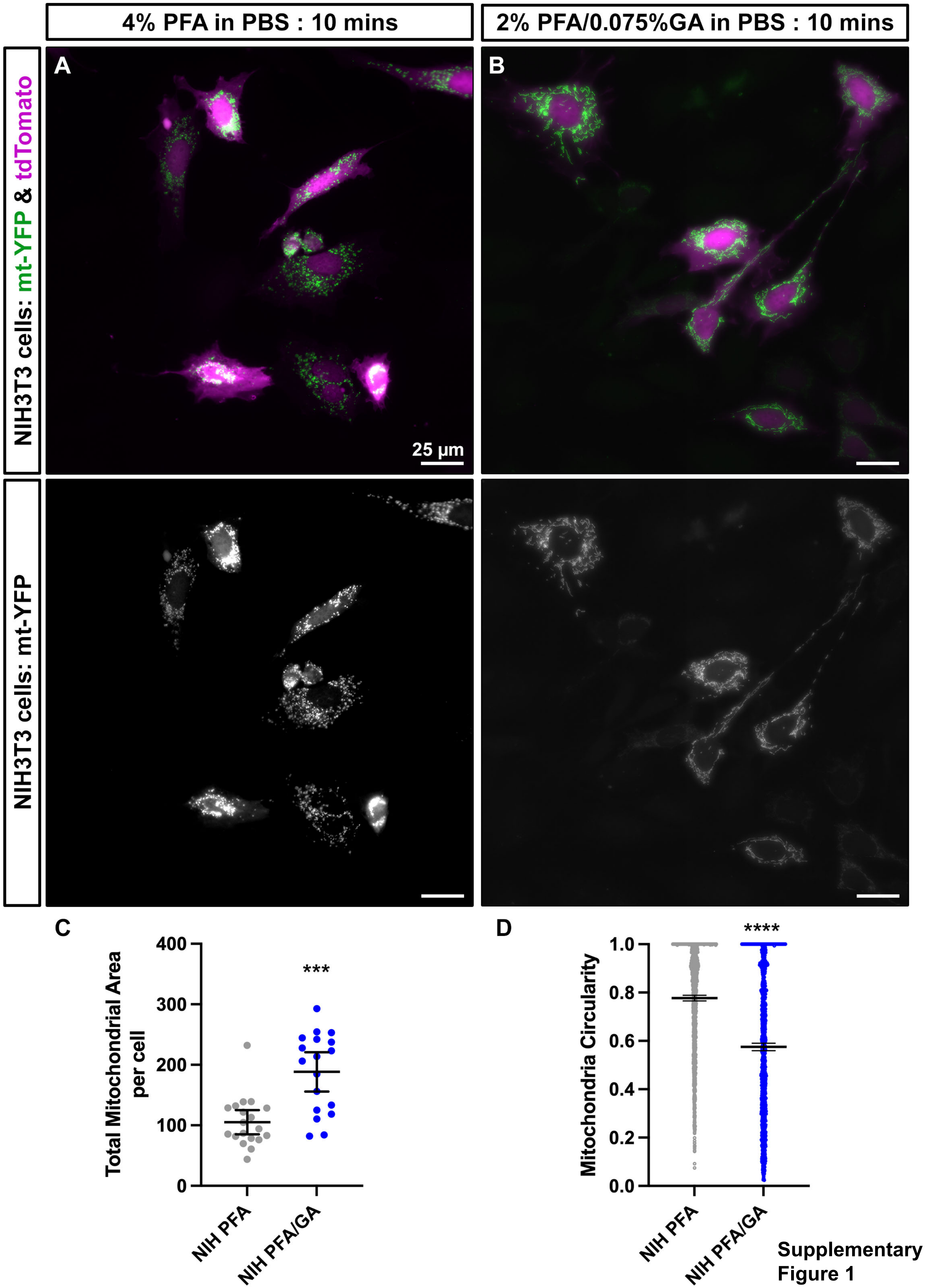

Supplementary Figure 1

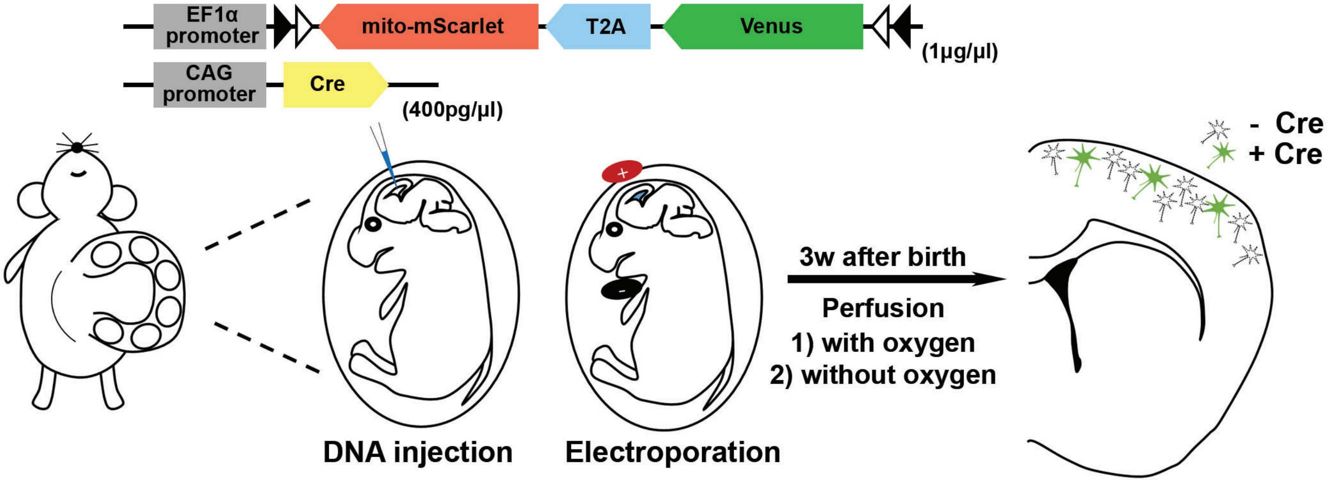

Supplementary Figure 2

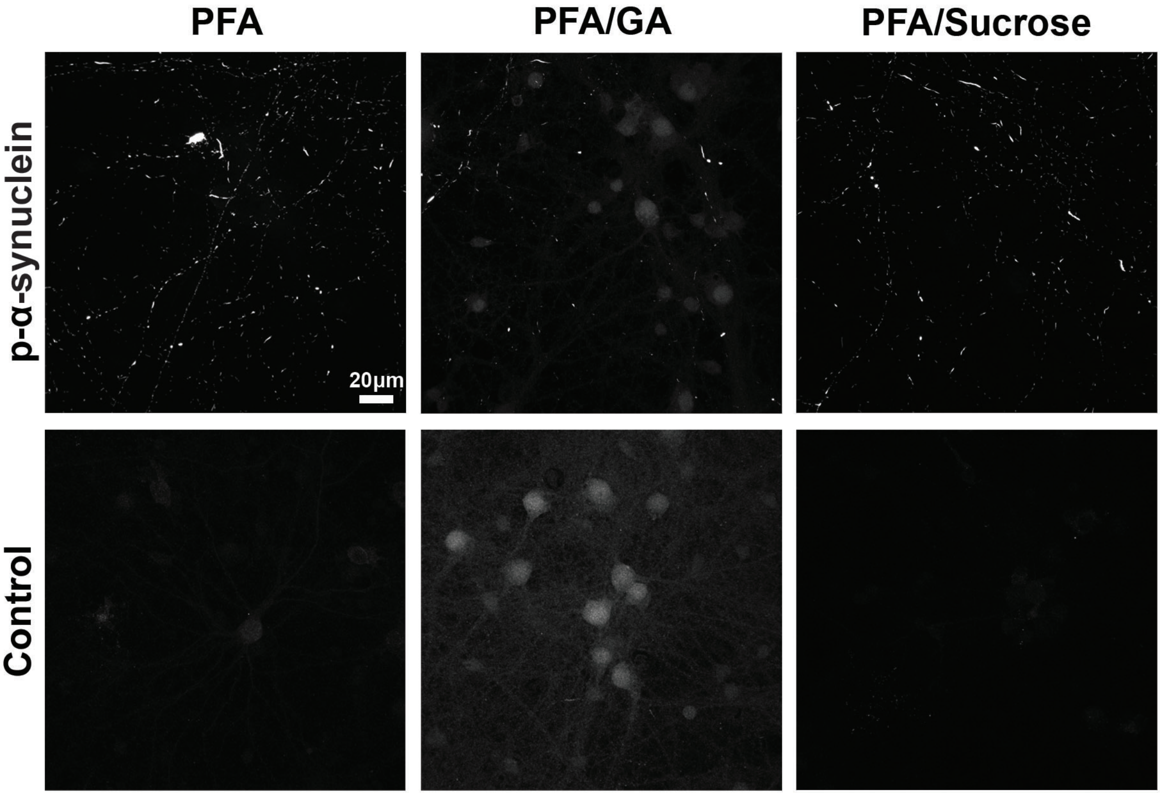

Supplementary Figure 3
